# The Protective Role of Attention-Deficit Hyperactivity Disorder Medication Against Post-disaster Psychopathology in Children: A Naturalistic Study After the 2023 Turkiye Earthquakes

**DOI:** 10.7759/cureus.101384

**Published:** 2026-01-12

**Authors:** Elif Kocak, Burak Kamis, Nazmiye Ince, Dilek Altun Varmis, Emine Merve Kalınlı, Emine Cilogullari, Kubra Sahin, Gupse Inal Ulutas, Tugce Hilda Demirci, Elif Gozde Yuce Antepuzumu, Cumali Yuksekkaya, Serkan Gunes

**Affiliations:** 1 Department of Child and Adolescent Psychiatry, Adana City Training and Research Hospital, Adana, TUR; 2 Department of Psychology, Toros University, Mersin, TUR

**Keywords:** attention deficit hyperactivity disorder (adhd), child and adolescent, clinical anxiety, clinical depression, natural disaster, post-traumatic stress disorder

## Abstract

Introduction: Children with attention-deficit hyperactivity disorder (ADHD) can be susceptible to psychological distress after traumatic events due to their difficulties in emotional regulation and stress reactivity. The present study explores psychiatric symptoms and the impact of pharmacological treatment in ADHD children following the 2023 Kahramanmaras earthquakes in Turkiye.

Methods: The study included 124 children diagnosed with ADHD and 58 controls without ADHD aged 8-12 years. ADHD children were divided into two subgroups: those who maintained ADHD medication post earthquake (ADHD-MED, N = 60) and those who discontinued it (ADHD-nonMED, N = 64). Psychological assessments included the Children’s Depression Inventory (CDI), State-Trait Anxiety Inventory (STAI), and Children’s Revised Impact of Event Scale-13 (CRIES-13).

Results: Significant differences were observed between the three groups in CDI, STAI, and CRIES-13 scores. The ADHD-nonMED group exhibited the highest scores of depressive, anxiety, and post-traumatic stress symptoms. In contrast, the ADHD-MED group showed intermediate scores, closer to those of the control group. The likelihood of clinical cut-off scores for severe anxiety and post-traumatic stress disorder was significantly greater in the ADHD-nonMED group compared to both the ADHD-MED and control groups.

Conclusions: Non-medicated ADHD children experienced more severe psychological symptoms after the earthquakes. Continued pharmacological treatment may have a protective role against post-disaster psychopathology in ADHD children.

## Introduction

Turkiye is located in a region with significant seismic activity, making it one of the most earthquake-prone countries in the world. On February 6, 2023, two powerful Kahramanmaras earthquakes struck Turkiye’s southeastern Anatolia region and impacted 11 cities [1]. These earthquakes along the East Anatolian Fault Zone led to widespread devastation, with over 50,000 fatalities reported in the region [2].

Natural disasters like the Kahramanmaras earthquakes are powerful and unpredictable events that severely harm people and the environment. Following a disaster, most victims require advanced medical attention for multiple and complex health issues. Psychiatric problems, including post-traumatic stress disorder (PTSD), anxiety disorders, and depression, are commonly observed after these major traumatic experiences [3,4].

Attention-deficit hyperactivity disorder (ADHD) is a prevalent neurodevelopmental condition marked by symptoms of impulsivity, inattention, and hyperactivity [5]. Children with ADHD frequently demonstrate poor emotional regulation, impaired executive functioning, and heightened stress sensitivity [5,6]. These features can place them at an elevated risk for developing psychiatric problems after traumatic events [7,8]. However, research on the psychiatric symptoms of ADHD children in the context of natural disasters is limited. Moreover, the moderating effect of pharmacological treatment for ADHD on psychological problems following a disaster remains underexplored. Thus, the present study aims to evaluate and compare psychological outcomes of children with and without ADHD after the Kahramanmaras earthquakes. It also examines whether the use of ADHD medication is linked to reduced severity of psychiatric symptoms in the post-disaster period.

## Materials and methods

Sample

The study involved 124 children with an ADHD diagnosis and 58 non-ADHD controls. All children were physically present in the disaster area during the earthquakes and experienced direct exposure, including structural collapse, emergency evacuation, injury, or the loss of a family member. Although detailed statistical comparisons regarding specific exposure types (e.g., rate of house damage or bereavement) were not performed, all groups were drawn from the same disaster-stricken region and clinical population, suggesting a broadly similar level of environmental stress exposure. They were all referred to Adana City Research and Training Hospital’s Child and Adolescent Psychiatry Clinic (ARTHCAP) between August 6 and December 6, 2023.

Children were included in the ADHD group based on the following criteria: (1) aged 8-12 years; (2) intelligence quotient (IQ) score greater than 80 (suggesting normal cognitive functioning); (3) ADHD diagnosis before the earthquakes; (4) no diagnosis of bipolar disorder, psychotic disorders, neurodevelopmental disorders other than ADHD, or any depressive or anxiety disorders prior to the earthquake; and (5) no diagnosis of chronic neurological or medical illnesses. Inclusion criteria for the control group were as follows: (1) aged 8-12 years; (2) IQ score greater than 80; (3) no diagnosis of bipolar disorder, psychotic disorders, neurodevelopmental disorders, or any depressive or anxiety disorders prior to the earthquake; and (4) no diagnosis of chronic neurological or medical illnesses.

Children were excluded from the study if they met any of the following criteria: (1) diagnosis of any comorbid neurodevelopmental disorder other than ADHD (e.g., autism spectrum disorder and intellectual disability) based on the Diagnostic and Statistical Manual of Mental Disorders, Fifth Edition (DSM-5) criteria; (2) diagnosis of psychotic disorders, bipolar disorder, or any depressive or anxiety disorders prior to the earthquake; (3) presence of any chronic neurological condition (e.g., epilepsy) or severe medical illness requiring ongoing treatment; and (4) missing or inaccessible medical records that prevented the verification of the prior ADHD diagnosis or medical history.

A total of 215 children were initially assessed for eligibility. Of these, 33 were excluded for not meeting the inclusion criteria. The final sample comprised 124 children in the ADHD group and 58 controls who met all study requirements.

Procedure

All children underwent thorough psychiatric assessments conducted by a child and adolescent psychiatry expert based on the DSM-5 criteria [9]. The assessment process included detailed clinical interviews with children and parents, behavioral observations, reviews of official medical records, and administrations of standardized rating scales. Additionally, a qualified psychologist applied the Wechsler Intelligence Scale for Children-Revised (WISC-R) [10,11] to evaluate intellectual capacities. Children diagnosed with any DSM-5 neurodevelopmental disorders aside from ADHD, psychotic disorders, or bipolar disorder were excluded from participation in the study.

The ADHD group included children diagnosed with ADHD before the earthquakes, with their diagnosis confirmed by a child and adolescent psychiatry expert at ARTHCAP. The verification of prior ADHD diagnosis was carried out through parent reports, clinical interviews, and medical records. Children with inaccessible medical records and unconfirmed ADHD diagnoses were excluded from the ADHD group.

Following the psychiatric evaluations, participants were instructed to complete the questionnaires. Instruments were administered in a standardized, quiet clinical setting with supervision to ensure comprehension. The Children’s Depression Inventory (CDI) [12], State-Trait Anxiety Inventory (STAI) [13], and Children’s Revised Impact of Event Scale-13 (CRIES-13) [14] were completed by children, and the Turgay DSM-IV Disruptive Behavior Disorders Rating Scale (T-DSM-IV-S) [15] and Conners’ Parent Rating Scale (CPRS) [16] were completed by parents.

Medication status

All children in the ADHD group were on medication prior to the earthquakes. After the disaster, they were categorized based on medication continuity into two groups: ADHD-MED (those who maintained their medication) and ADHD-nonMED (those who discontinued it). The main reasons why ADHD children could not receive medication after the earthquakes were difficulties accessing medication. Many people prioritized basic needs such as shelter and safety over psychiatric care, while others faced emotional or financial hardship.

The medication status was verified through caregiver reports, supported by electronic prescriptions, follow-up notes, and pharmacy data. The children who continued pharmacological treatment received methylphenidate (MPH) and/or atomoxetine (ATX). The dosage of MPH varied between 20 and 40 mg/day, while ATX was prescribed at doses between 40 and 60 mg/day. These dosage ranges are consistent with the standard clinical guidelines and therapeutic recommendations for children in this age group.

Instruments

The Turkish versions of all scales were administered to the participants, and the validated forms were used for scoring.

Children’s Depression Inventory

To evaluate the severity of depressive symptoms, the Children’s Depression Inventory (CDI) was employed [12]. This self-report scale consists of 27 items, each addressing a specific symptom of depression. For every item, the child selects one of three statements (scored 0-2) that best reflects their feelings over the preceding two weeks [17]. Total scores range from 0 to 54, with higher values denoting greater symptom severity. A cut-off score of 25 is widely used to identify potential depression [17]. The Turkish adaptation and validity study of the CDI was performed by Oy [18].

State-Trait Anxiety Inventory

Anxiety levels were assessed using the State-Trait Anxiety Inventory (STAI), a standardized self-report measure that distinguishes between temporary (state) and enduring (trait) anxiety [13]. The State Anxiety subscale (S-STAI) captures the individual's current emotional status, while the Trait Anxiety subscale (T-STAI) evaluates general anxiety disposition. Comprising 20 items rated on a 1-4 scale, the STAI yields scores ranging from 20 to 80. A cut-off score of 39-40 is commonly used to indicate a clinically significant anxiety [13]. For this study, the Turkish version of the STAI was utilized, and a score of 40 was adopted as the threshold for identifying significant anxiety symptoms [19].

Children’s Revised Impact of Event Scale-13

The risk of PTSD was screened using the Children’s Revised Impact of Event Scale-13 (CRIES-13) [14]. This tool asks children to rate the frequency of trauma-related symptoms experienced during the past week on a scale from 0 (none) to 5 (a lot). The scale comprises 13 items divided into three subscales: intrusion (four items), avoidance (four items), and arousal (five items). Total scores can range from 0 to 65, with a score of 30 or higher suggesting a high likelihood of PTSD [14]. The Turkish adaptation of the scale has been validated by Ceri et al., confirming its reliability and validity [20].

Turgay DSM-IV Disruptive Behavior Disorders Rating Scale

ADHD symptoms were evaluated using the Turgay DSM-IV Disruptive Behavior Disorders Rating Scale (T-DSM-IV-S). Developed by Turgay [15] and adapted for the Turkish population by Ercan et al. [21], this instrument is grounded in DSM-IV criteria. It screens for hyperactivity, impulsivity, inattention, oppositional defiant disorder, and conduct disorder. Parents rate items on a 0-3 point scale [15]. Although based on previous diagnostic criteria, this scale remains the standard and most widely used validated instrument in Turkiye. Given the high concordance of core ADHD symptoms with the current DSM-5, it continues to be a valid tool for monitoring symptom severity in the current clinical context. For the purpose of this study, only the inattention and hyperactivity-impulsivity subscales were utilized to support the ADHD diagnosis.

Conners’ Parent Rating Scale

Behavioral issues were further assessed using the Conners’ Parent Rating Scale (CPRS) [16]. This 48-item questionnaire utilizes a Likert-type scoring system to measure five domains: conduct problems, learning difficulties, psychosomatic complaints, impulsivity-hyperactivity, and anxiety [16]. The Turkish validation study by Dereboy et al. demonstrated the scale's applicability to Turkish children [22]. In this study, the CPRS was used as an ancillary tool to corroborate the clinical diagnosis of ADHD.

Statistical analysis

All statistical computations were performed using the IBM SPSS Statistics software, version 22.0 (IBM Corp., Armonk, NY). The demographic and clinical characteristics of the study population were presented using descriptive statistical methods. To determine whether the continuous variables followed a normal distribution, the Kolmogorov-Smirnov and Shapiro-Wilk tests were utilized. For quantitative variables that demonstrated a normal distribution, comparisons across the three groups were conducted using the one-way analysis of variance (ANOVA), while pairwise comparisons were analyzed via the independent samples t-test. Differences in categorical variables were evaluated using the chi-square (χ²) test. For all analyses, a p-value of less than 0.05 was established as the threshold for statistical significance.

Ethical consideration

The study adhered strictly to the ethical guidelines outlined in the Declaration of Helsinki. Approval for the research protocol was granted by the Ethics Committee of Adana City Training and Research Hospital. Prior to participation, the purpose and methods of the study were clearly communicated to all families. Written informed consent was provided by the parents or legal guardians, and assent was obtained from all participating children.

## Results

The simple clinical profile of the study participants is summarized in Table 1. The mean age of the ADHD-MED group was 10.02 ± 1.28 years, with females comprising 45.0% (N = 27) of the sample. Children in the ADHD-nonMED group had a mean age of 10.05 ± 1.32 years, and 45.3% (N = 29) were females. The mean age in the control group was 9.95 ± 1.36 years, and 44.8% of the group (N = 26) were females. There were no significant differences in age and gender between the groups (p > 0.05). In the ADHD-MED group, 81.7% (N = 49) of the children were receiving MPH, 13.3% (N = 8) were on ATX, and 5% (N = 3) were treated with a combination of MPH and ATX.

**Table 1:** Characteristics of the study sample. P-values were obtained using one-way ANOVA for age and chi-square test (χ²) for gender. ADHD: attention-deficit hyperactivity disorder; MPH: methylphenidate; ATX: atomoxetine.

Characteristics		ADHD-MED, N = 60	ADHD-nonMED, N = 64	Controls, N = 58	p
Age (mean, SD)	Years	10.02 (1.28)	10.05 (1.32)	9.95 (1.36)	0.920
Gender (N, %)	Male	33 (55.0)	35 (54.7)	32 (55.2)	0.999
	Female	27 (45.0)	29 (45.3)	26 (44.8)	-
Medication	MPH	49 (81.7)	-	-	-
	ATX	8 (13.3)	-	-	-
	MPH+ATX	3 (5.0)	-	-	-

**Figure 1:**
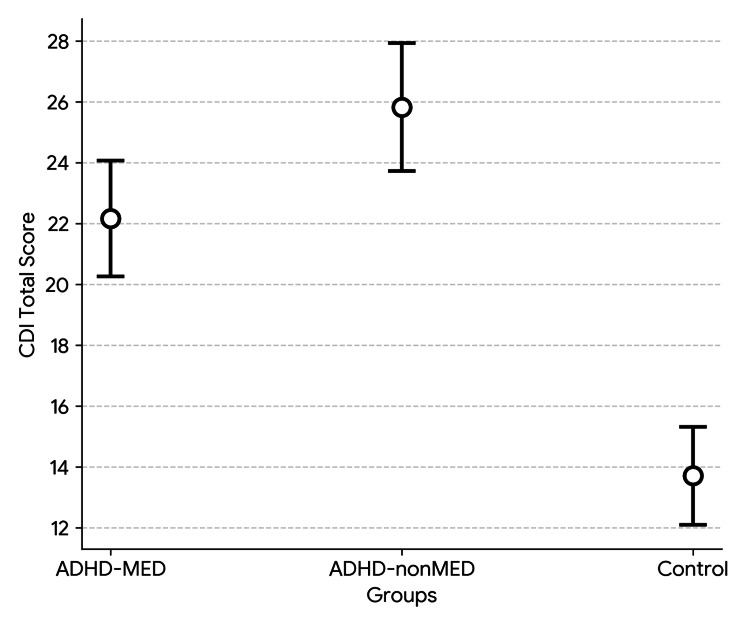
CDI scores between the study groups. ADHD: attention-deficit hyperactivity disorder; CDI: Children’s Depression Inventory.

**Figure 2:**
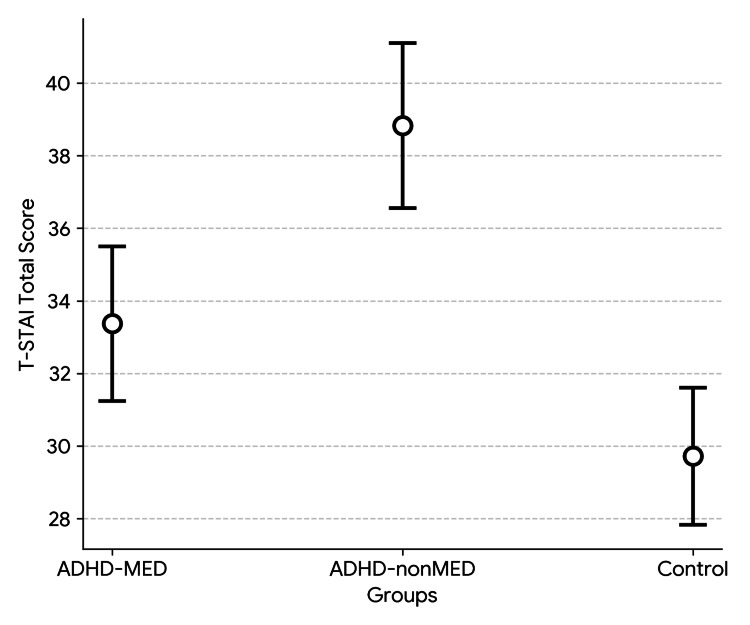
T-STAI scores between the study groups. ADHD: attention-deficit hyperactivity disorder; T-STAI: Trait Anxiety subscale of State-Trait Anxiety Inventory.

**Figure 3:**
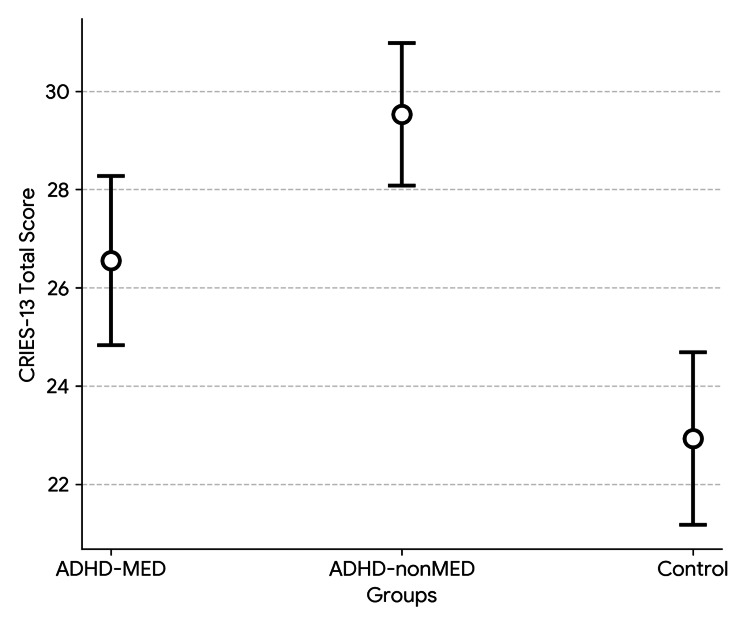
CRIES-13 scores between the study groups. ADHD: attention-deficit hyperactivity disorder; CRIES-13: Children’s Revised Impact of Event Scale-13.

Table 2 presents a comparison of CDI, T-STAI, and CRIES-13 scores across the three groups (Figures 1-3). As shown in the table, significant differences were found between the groups in the CDI total score, T-STAI total score, and CRIES-13 total score, as well as the intrusion and avoidance subscores of the CRIES-13. Additional analyses were carried out to determine the specific groups responsible for the significant differences. All two-group comparisons showed significant differences, except for the avoidance subscore. The significant difference in the avoidance subscore was attributable to the ADHD-nonMED group, as no meaningful difference was observed between the ADHD-MED and control groups (p = 0.502). In the binary comparison between the ADHD-MED and ADHD-nonMED groups, the ADHD-nonMED group demonstrated significantly higher scores on the CDI total (p = 0.013), T-STAI total (p = 0.003), CRIES-13 total (p = 0.010), and intrusion subscore (p = 0.001).

**Table 2:** The comparison of CDI, T-STAI, and CRIES-13 scores between the groups. P-values were obtained using one-way ANOVA. ADHD: attention-deficit hyperactivity disorder; CDI: Children’s Depression Inventory; T-STAI: Trait Anxiety subscale of State-Trait Anxiety Inventory; CRIES-13: Children’s Revised Impact of Event Scale-13.

Clinical scales	ADHD-MED, N = 60, Mean (SD)	ADHD-nonMED, N = 64, Mean (SD)	Controls, N = 58, Mean (SD)	p
CDI Total	22.17 (7.52)	25.83 (8.60)	13.71 (6.26)	0.000
T-STAI Total	33.37 (8.43)	38.83 (9.28)	29.72 (7.34)	0.000
CRIES-13 Total	26.55 (6.81)	29.53 (5.93)	22.93 (6.82)	0.000
CRIES-13 Intrusion	12.32 (2.34)	13.86 (2.50)	9.09 (3.13)	0.000
CRIES-13 Avoidance	6.03 (2.32)	6.69 (1.85)	5.76 (2.10)	0.043
CRIES-13 Arousal	8.03 (2.71)	9.11 (2.25)	8.01 (3.11)	0.057

Table 3 presents the comparison of depression, severe anxiety, and PTSD likelihood rates across the study groups. As seen in the table, significant differences were observed in the rates of depression, severe anxiety, and PTSD across the groups. The additional analyses showed that the control group was the main source of the difference in depression rates. There was no meaningful difference between the ADHD-MED and ADHD-nonMED groups in terms of depression rates (p = 0.117). On the other hand, the differences in severe anxiety and PTSD rates were primarily attributable to the ADHD-nonMED group, as no significant differences were observed between the ADHD-MED and control groups (p = 0.210 and p = 0.137, respectively).

**Table 3:** The comparison of the likelihood of depression, anxiety, and PTSD between the groups. P-values were obtained using the chi-square (χ²) test. ADHD: attention-deficit hyperactivity disorder; PTSD: post-traumatic stress disorder; CDI: Children’s Depression Inventory; T-STAI: Trait Anxiety subscale of State-Trait Anxiety Inventory; CRIES-13: Children’s Revised Impact of Event Scale-13.

Clinical thresholds	ADHD-MED, N = 60, N (%)	ADHD-nonMED, N = 64, N (%)	Controls, N = 58, N (%)	p
CDI Total ≥ 25	13 (21.7)	21 (32.8)	4 (6.9)	0.002
CDI Total < 25	47 (78.3)	43 (67.2)	54 (93.1)	
T-STAI Total ≥ 40	15 (25.0)	26 (40.6)	10 (17.2)	0.013
T-STAI Total < 40	45 (75.0)	38 (59.4)	48 (82.8)	
CRIES-13 Total ≥ 30	14 (23.3)	24 (37.5)	8 (13.8)	0.010
CRIES-13 Total < 30	46 (76.7)	40 (62.5)	50 (86.2)	

Given that the vast majority of medicated children were receiving MPH, with only a few cases using ATX or a combination of both, conducting subgroup analyses based on medication type was not considered statistically appropriate due to insufficient power.

## Discussion

Previous research has highlighted that ADHD children tend to exhibit heightened reactivity to environmental stressors, difficulties in cognitive reframing of traumatic experiences, and challenges in maintaining behavioral regulation, all of which can exacerbate psychological distress in the aftermath of trauma [23,24]. In line with this, the current study demonstrated that non-medicated ADHD children exhibited significantly elevated levels of depressive, anxiety, and PTSD-related symptoms after traumatic exposure to the earthquakes. Conversely, medicated ADHD children showed intermediate symptom severity, more comparable to the control group. These findings may underscore the importance of recognizing ADHD as a risk factor for adverse psychological outcomes following natural disasters. Clinicians working in disaster zones should consider the proactive identification of at-risk ADHD children and ensure they receive adequate psychological support.

Although several studies have explored the impact of trauma on ADHD children, research specifically focusing on natural disasters remains limited. In a clinical sample of 104 adolescents with ADHD, lifetime depression was significantly associated with trauma exposure, independent of ADHD symptom severity [25]. A meta-analysis indicated significant associations between PTSD and externalizing psychopathologies like ADHD [26]. A five-year longitudinal study involving 1,829 detained adolescents found that ADHD symptoms significantly predicted the development of PTSD in male participants [27]. In a survey of the Wenchuan earthquake (China, 2008), ADHD and conduct disorder were concurrently linked with PTSD at both six and 18 months following the disaster [28]. Another earthquake study from Turkiye reported that while ADHD comorbidity was not a major predisposing factor for PTSD, it might contribute to its worsening after PTSD onset [29]. In our study, the absence of medication in ADHD children was associated with increased rates of clinically significant anxiety and PTSD after the earthquakes. These results can provide evidence for the benefits of continued ADHD pharmacotherapy in reducing the risks of post-disaster psychopathology. In this context, disaster response strategies should incorporate mental health continuity plans. These may include pre-arranged telemedicine services, mobile psychiatric teams, and policies ensuring rapid access to essential psychotropic medications even during crisis periods.

The lower psychological distress in medicated ADHD children likely reflects mechanisms beyond the core management of ADHD symptoms. Commonly prescribed ADHD medications such as MPH and ATX may also enhance emotional regulation and stress resilience, which are critical capacities in post-traumatic adaptation [30,31]. MPH increases dopaminergic and noradrenergic activity in the prefrontal cortex, which may support executive control over intrusive or dissociative trauma responses [32]. Similarly, ATX, a selective norepinephrine reuptake inhibitor, has demonstrated anxiolytic properties in pediatric ADHD populations, potentially buffering against trauma-related anxiety [33]. The pharmacological effects of these medications could underlie a more regulated and organized emotional response to disaster-related stress.

Despite the strengths of this study, several limitations must be noted. A major limitation is the cross-sectional design that precludes causal interpretations. While group differences in psychological outcomes are observed, it is not possible to determine with certainty whether medication continuity directly mitigates symptom severity. Non-pharmacological treatments like behavioral therapy, psychoeducation, or family support services were not systematically assessed. These interventions may significantly influence post-traumatic outcomes and interact with pharmacological treatment. Confounding variables, such as parental psychopathology, family functioning, socioeconomic status, or trauma severity, were not controlled or quantified. Additionally, the primary outcome measures (CDI, STAI, and CRIES-13) relied solely on child self-reports. While these are validated instruments, the absence of multi-informant assessments (e.g., teacher or parent reports for these specific symptoms) may limit the objectivity of the findings. Longitudinal follow-up studies evaluating multiple therapeutic options and confounding factors are needed to better understand the psychological responses of ADHD children after mass disasters.

## Conclusions

The present study provides controlled empirical evidence highlighting the vulnerability of children with ADHD in the aftermath of natural disasters, specifically earthquakes. Our findings demonstrate that unmedicated ADHD children are at a significantly higher risk for developing severe depressive, anxiety, and post-traumatic stress symptoms compared to their medicated peers and neurotypical controls. This disparity suggests that the continuity of pharmacological treatment plays a critical protective role, extending beyond the management of core ADHD symptoms to the mitigation of secondary post-disaster psychopathology.

Scientifically, this protective effect may be attributed to the capacity of ADHD medications, such as MPH and ATX, to enhance emotional regulation and stress resilience. By increasing dopaminergic and noradrenergic activity in the prefrontal cortex, these treatments likely support executive control over intrusive trauma responses, thereby preventing the escalation of psychological distress. Consequently, the interruption of treatment due to logistical failures during disasters may leave these children defenseless against the overwhelming stress of trauma. In light of these results, disaster response strategies must evolve to recognize ADHD not merely as a behavioral disorder but as a significant risk factor for trauma-related psychiatric morbidity. Future disaster management policies should prioritize the rapid re-establishment of psychiatric supply chains and the deployment of mobile mental health teams to ensure treatment continuity. Clinicians operating in disaster zones should proactively identify children with ADHD to provide immediate pharmacological and psychological support, thereby fostering better long-term adaptation and reducing the burden of post-traumatic stress.

## Appendices

Sociodemographic and clinical data form

A. Sociodemographic Information

1. Age: _______

2. Gender:

( ) Female

( ) Male

3. Education Level/Grade: _______

B. Clinical History (ADHD)

4. Age at ADHD Diagnosis: _______

5. Medication Status Prior to Earthquake:

( ) Yes (Specify drug and dose: _______________)

( ) No

6. Medication Status After Earthquake:

( ) Continued Medication (ADHD-MED group)

( ) Discontinued Medication (ADHD-nonMED group). Reason for discontinuation: ___________________

C. Earthquake Exposure Details

7. Proximity to Earthquake Epicenter:

( ) Within the affected 11 cities

( ) Outside the region

8. Type of Trauma Exposure (Check all that apply):

( ) Structural collapse/Trapped under rubble

( ) Emergency evacuation from home

( ) Physical injury sustained during the earthquake

( ) Loss of a first-degree family member

( ) Witnessing the death or severe injury of others

( ) Significant property damage (home uninhabitable)
